# The importance of Fcγ and C-type lectin receptors in host immune responses during *Pneumocystis* pneumonia

**DOI:** 10.1128/iai.00276-24

**Published:** 2024-12-31

**Authors:** Theodore J. Kottom, Eva M. Carmona, Kyle Schaefbauer, Kimberly E. Stelzig, Madeline R. Pellegrino, Marc Bindzus, Andrew H. Limper

**Affiliations:** 1 Department of Internal Medicine, Division of Pulmonary and Critical Care Medicine, the Thoracic Diseases Research Unit, Mayo Clinic College of Medicine, Rochester, Minnesota, USA; NIH, NIAID, Washington, DC, USA

**Keywords:** Pneumocystis carinii, inflammation, clearance, Fc receptors, C-lectin receptor

## Abstract

*Pneumocystis jirovecii* pneumonia (PJP) remains a significant cause of morbidity and mortality during AIDS. In AIDS, the absence of CD4 immunity results in exuberant and often fatal PJP. In addition, organism clearance requires a balanced macrophage response since excessive inflammation promotes lung injury and respiratory failure. Corticosteroids given in addition to antibiotics significantly improve outcomes during PJP. However, concerns exist that corticosteroids further suppress immunity and increase co-infections. New strategies to promote killing and clearance of *Pneumocystis* while balancing lung inflammation are required. Prior studies have shown that innate immunity to *Pneumocystis* is mediated by C-type lectin receptors (CLRs) on macrophages and involves downstream CARD9 activation. CARD9 can be targeted by a novel specific small molecule inhibitor (BRD5529) that significantly reduces inflammatory signaling by macrophages. CARD9 serves as the central intracellular molecule through which Dectin-1, Dectin-2, Mincle, and other CLRs signal. Dectin-1 CLR is activated through its own intracytoplasmic domain, whereas other innate CLRs (e.g., Dectin-2 and Mincle) require interactions with a common Fc-gamma receptor (FcγR) accessory chain to mediate responses. We now observe that mice double deficient in both Dectin-1 and Fcer1g (which lack the FcγR gamma chain) exhibit markedly reduced organism clearance compared with *Card9*^−/−^ infected animals. These mice also possess deficiencies in immunoglobulin (Ig) Fc receptors directly mediating antibody responses, further implicating altered humoral responses in *Pneumocystis* killing. We further demonstrate in the *Pneumocystis* pneumonia (PCP) mouse model that BRD5529 administration successfully suppresses inflammatory cytokines. Our data support that innate immune responses through the CLR-CARD9 axis and humoral response act together to mediate effective responses resulting in optimal organism killing and generation of host inflammatory responses. Furthermore, host lung inflammation during PCP may be successfully reduced with a novel CARD9 small molecule inhibitor.

**IMPORTANCE:**

*Pneumocystis* pneumonia (PCP) causes severe respiratory impairment in hosts with suppressed immunity, particularly those with CD4 deficiencies, such as HIV. In addition to lymphocytic immunity, both innate and humoral immunities also participate in host defense against *Pneumocystis*. In the current studies, we defined the relative roles of CLR receptor-mediated inflammation, as well as FcgR-related inflammation and clearance of *Pneumocystis* organisms. Our studies reveal important roles for CLR activities for inducing lung inflammation, which can be ameliorated with a novel small molecule inhibitor of the CARD9 adaptor protein that is necessary for CLR signaling. In contrast, FcgR has a dominant role in organism clearance, underscoring an integral role of humoral responses for the elimination of this infection.

## INTRODUCTION

On the 43th anniversary of the AIDS/HIV pandemic, *Pneumocystis jirovecii* pneumonia (PJP) remains a serious continuing concern to those with AIDS. PJP represents a major health threat, with over 400,000 cases worldwide each year (1). In regions with lower resources, such as Sub-Saharan Africa, PJP outcomes continue to remain poor (2). Depending on underlying host factors, the case mortality rates for PJP patients can be as high as ~40% (3). Generally, the clearance of *Pneumocystis* requires intact CD4 immunity (4). However, in conditions of lymphocytic impairment, such as during AIDS, organisms thrive, resulting in uncontrolled pneumonia (5, 6). In addition, *Pneumocystis* organism clearance also requires optimal macrophage activity (7). Macrophage activation upon binding to *Pneumocystis* not only results in host inflammatory response via the CLR-CARD9 pathway (8), which is needed for organism clearance, but can also promote lung injury during severe *Pneumocystis* pneumonia (PCP) (9, 10). For example, the release of *Pneumocystis* β-glucans during the initiation of antibiotic therapy often exacerbates this response and can lead to initial clinical deterioration during PJP therapy (11, 12). Our prior work further demonstrated that macrophage polarization toward M1 phenotype in PCP causes significant detrimental host inflammation during infection (13). Accordingly, anti-inflammatory corticosteroids, administered concurrently with antibiotics, can reduce host inflammation and improve outcomes (14). Nevertheless, steroid-induced immunosuppression also increases the risk of secondary coinfections (15, 16).

In addition to functional CD4 immunity, others have shown important roles for humoral immune factors in the control of *Pneumocystis* (4, 17). Antibody opsonization of organisms strongly augments clearance by phagocytic cells. Although the role of antibody opsonization is not well studied in pathogenic fungi, some studies have provided important insights including cases of PJP in patients receiving B-cell depletion (18), murine studies documenting the roles of CD40 on B cell immunity during pneumonia (19, 20), and observations that passive antibody treatment with anti-*Pneumocystis* antibody can provide PCP prophylaxis in rodents (21). Thus, new strategies promoting recognition, killing, and clearance of *Pneumocystis* and newer adjuvant anti-inflammatory treatment options are needed for the optimal treatment of PCP.

Our prior studies have shown the vital importance of the CARD9 pathway along with the upstream CLRs Dectin-1 and Mincle, which interact with β-glucans and the major surface glycoproteins, respectively, in mediating the host responses and organism clearance during PCP (8, 22–24). In addition, we have shown that other CLR family members CLEC12A, DC-SIGN, MCL, MGL-1, and SIGNR3 (mouse DC-SIGN homolog) also bind *Pneumocystis* cell wall components and may be important for host immune response and organism killing (25, 26). Furthermore, we have published that the small molecule BRD5529 that binds CARD9 protein can significantly inhibit downstream-mediated inflammatory signaling in macrophages as well as in mouse lungs stimulated with fungal β-glucans (27, 28). Together, these important parallel pathways provide new opportunities for the development of novel treatment strategies for patients with PCP refractory to antibiotics alone.

## RESULTS

### Dectin-1/Fcγ-deficient mice display significantly greater organism burdens compared with CD4-deficient mice with PCP

**Fig 1:**
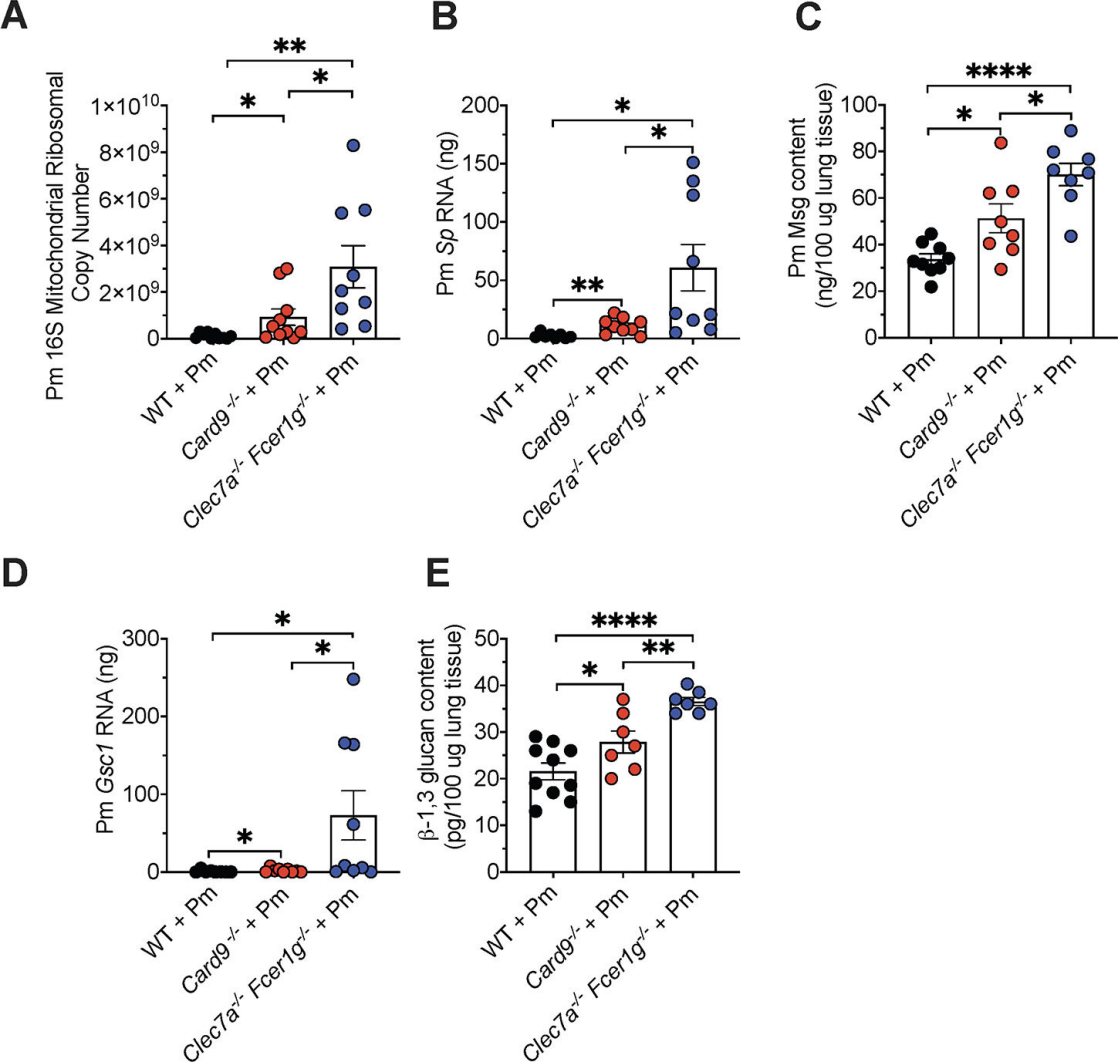
*Clec7a*^−/−^ *Fcer1g*^−/−^ mice exhibit significantly increased organism burden during PCP. *Card9*^−/−^, *Clec7a*^−/−^ *Fcer1g*^−/−^, and WT mice were immunosuppressed by the depletion of CD4 cells and subsequently inoculated with *P. murina* (Pm) organisms. (**A**) After 8 weeks, the Pm organism burden was determined by Pm *16S mitochondrial ribosomal* DNA copy number and assessed using a standard curve. Data are derived from 7 to 10 mice per group ± SEM; *P < 0.05,**P < 0.01, ***P < 0.001, and ****P < 0.0001 comparing Pm burden in the *Card9*^−/−^, *Clec7a*^−/−^ *Fcer1g*^−/−^, and WT mice and are representative of two separate experiments. (**B**) qPCR analysis of trophic-specific *Sp* RNA. (**C**) ELISA analysis of the major surface glycoprotein (Msg) from the respective infected lung tissues. (**D**) qPCR analysis of cyst-specific *Gsc1* RNA. (**E**) ELISA analysis of the β−1,3 glucan content from the respective infected lung tissues.

**Fig 2:**
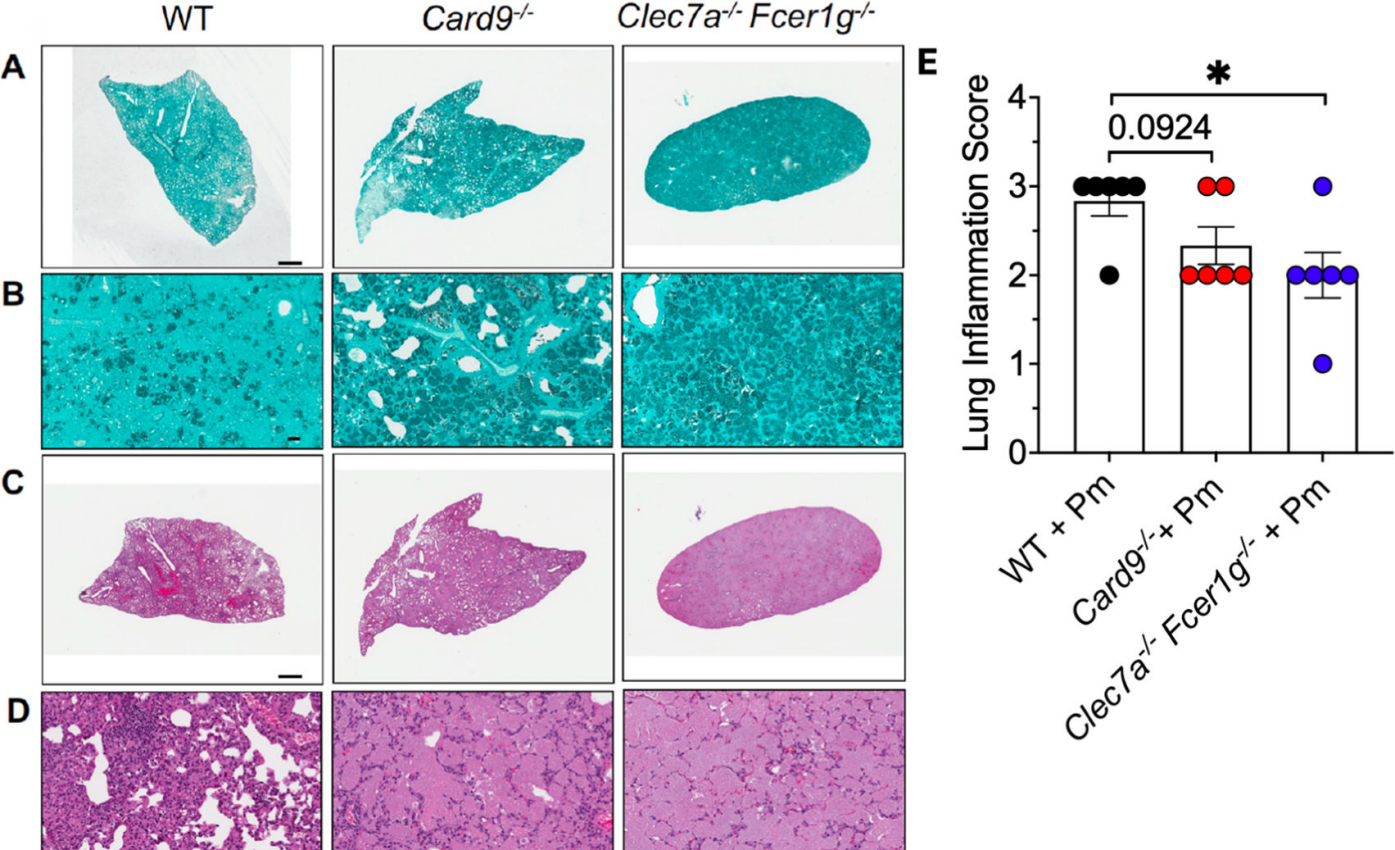
Analysis of lung sections shows exuberant cyst forms in the *Clec7a*^−/−^ *Fcer1g*^−/−^ mice and less inflammation. After 8 weeks of infection, the lungs were fixed in 10% phosphate-buffered formalin, and 5 µm sections were obtained. Gomori Methenamine Silver stains (GMS) for *P. murina* (Pm) organisms were performed, demonstrating numerous more cyst life forms in the *Clec7a*^−/−^ *Fcer1g*^−/−^ mice lungs (A and B, panel 3) versus WT (A and B, panel 1) and *Card9*^−/−^ lungs (A and B, panel 2). H&E staining showed the presence of substantially fewer lymphocytic aggregates in the *Clec7a*^−/−^ *Fcer1g*^−/−^-infected PCP mouse lung (C and D, panel 3) compared with the other two mouse groups. As indicated, for original magnification, ×70, scale bars are 1,500 µm (**A and C**) and for original magnification ×400, scale bars are 25 µm (**B and D**). Quantification of H&E slides with a lung inflammation score (**D**). *P < 0.05, comparing Pm-infected WT, *Card9*^−/−^, and *Clec7a*^−/−^ *Fcer1g*^−/−^ mice. The data shown are derived from six animals and shown as mean ± SEM per group.

We and others have shown that mice with CARD9 deficiency are significantly susceptible to fungal infections (8, 29). In the case of PCP, this is associated with increased *Pneumocystis murina* organism burdens and reduced inflammatory responses (8). Interestingly, others have shown that phenocopying the CARD9 deficiency using a Dectin-1/Fcγ subunit knockout mouse (termed *Clec7a*^−/−^*Fcer1g*^−/−^) resulted in similar neutrophil recruitment and fungal burdens in the brain in a central nervous system (CNS) *Candida albicans* model compared with CARD9 deficiency (30). Therefore, we tested *Clec7a*^−/−^*Fcer1g*^−/−^ compared with *Card9^−/−^* and wild-type (WT) mice in the mouse CD4-deficient PCP model, which mimics CD4 impairment during AIDS. Although *Card9^−/−^* displayed markedly increased *P. murina* (Pm) burdens compared with WT infection mice as we published previously (8), we further noted even significantly greater *P. murina* burdens in the *Clec7a*^−/−^*Fcer1g*^−/−^ PCP mouse model quantitated by qPCR (Fig. 1A). The analysis of life-form-specific qPCR markers, including those for the trophic form (Sp), a serine protease expressed approximately 8-fold more in this life form, and Gsc1, which encodes a β−1,3-glucan synthase highly expressed in the cyst form (31), revealed a significantly higher abundance of these *Pneumocystis* forms in the *Clec7a^−/−^Fcer1g^−/−^* infected lungs compared with the other two mouse strains tested (Fig. 1B and D). In addition, we implemented Enzyme Linked ImmunoSorbant Assay (ELISA) and lung β−1,3 glucan analysis methods and showed that *Clec7a*^−/−^*Fcer1g*^−/−^ infected mice had higher major surface glycoprotein (more prominent on trophic life forms) (32) as well as greater total β−1,3 glucan (cyst specific carbohydrate) (27, 33, 34) than either the *Card9^−/−^* or the WT mice in the immunosuppressed model (Fig. 1C and E). Silver staining on lung sections of *Clec7a*^−/−^*Fcer1g*^−/−^ *P. murina*-infected mice revealed the presence of significantly more cysts (Fig. 2A, panel 3) in comparison to their WT and *Card9*^−/−^-infected counterparts. Higher magnification (Fig. 2B, panel 3) of the *Clec7a*^−/−^ *Fcer1g*^−/−^ mouse lung illustrates this abundance of cystic forms. Hematoxylin and eosin (H&E) staining of the three respective mouse lungs revealed the presence of abundant inflammatory cell aggregates in the WT-infected groups compared with the other two infected groups (Fig. 2C and D, panel 1). H&E lung inflammation slide scoring of the respective animal groups showed a significantly higher inflammatory scores in the WT-infected animals compared with the *Clec7a*^−/−^*Fcer1g*^−/−^ mice. *Card9^−/−^* animals also showed less severe lung inflammation compared with the WT animals (*P* = 0.0924) (Fig. 2E). In addition, the overall lung cellularity of the *Card9^−/−^* mice with *Pneumocystis* pneumonia was significantly reduced compared with the wildtype-infected animals, and the *Clec7a*^−/−^*Fcer1g*^−/−^ mice with PCP exhibited even greater reductions in overall lung cellularity (Fig. S1). We also observed, through H&E staining, that the *Clec7a*^−/−^*Fcer1g*^−/−^ mice exhibited greater amounts of intra-alveolar proteinaceous exudates rich in organisms (previously described and observed in the H&E slides [35]) and some evidence of alveolar damage compared with the other strains examined. Despite substantial differences in inflammation and organism burdens, over the time course of these experiments, we did not observe any differences in mortality between the various strains tested.

### Dectin-1/Fcγ knockout mice demonstrate suppressed host cytokine responses during *Pneumocystis* pneumonia

**Fig 3:**
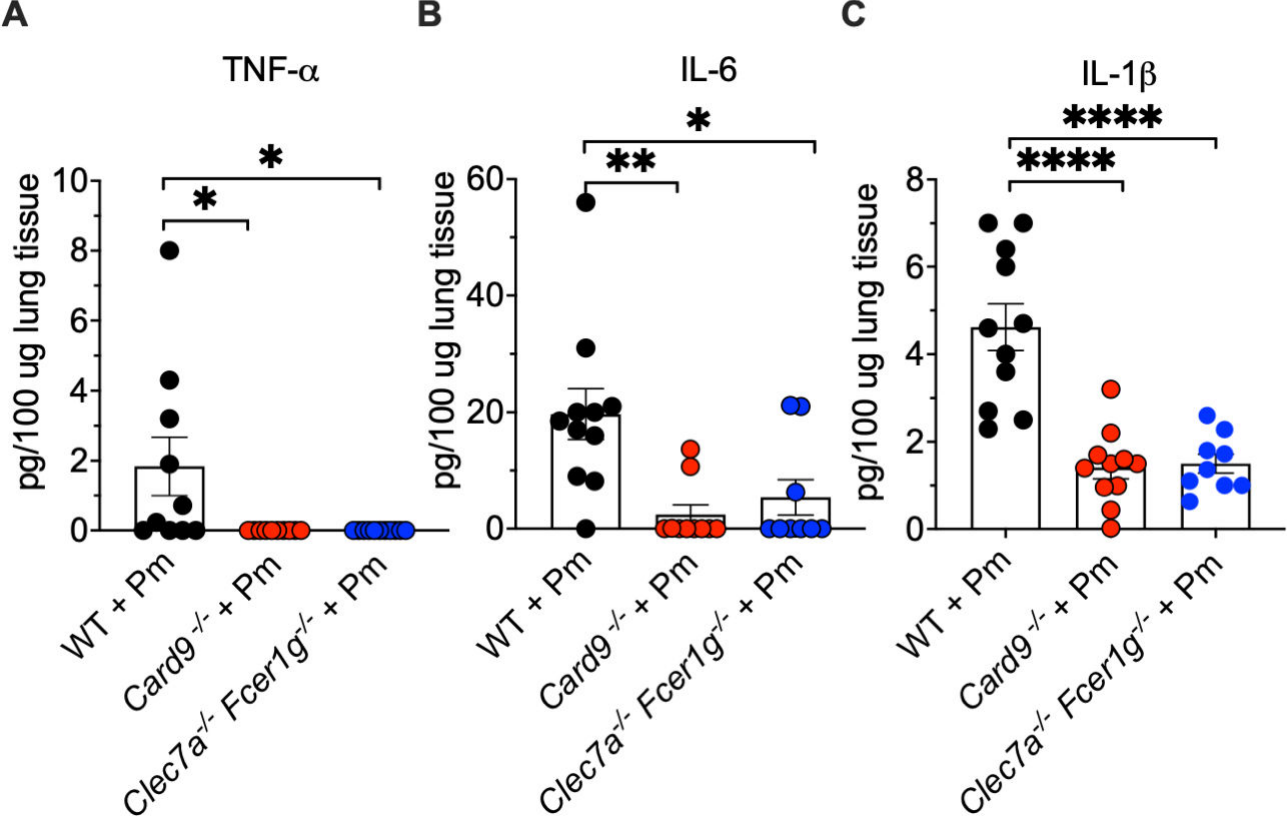
Inflammatory cytokines are significantly reduced in the lungs of *Clec7a*^−/−^ *Fcer1g*^−/−^ PCP-induced mice in absence. WT, *Clec7a*^−/−^ *Fcer1g*^−/−^, or *Card9*^−/−^ mice were infected with Pm. After 8 weeks of infection, the lungs were harvested, and protein lysates were obtained. Levels of the indicated inflammatory cytokines were measured using ELISA. *P < 0.05, **P < 0.01, and ****P < 0.0001 comparing Pm infected WT vs *Card9*^−/−^ and *Clec7a*^−/−^ *Fcer1g*^−/−^ mice, and the data shown are derived from *n* > 9 animals and shown as mean ± SEM per group and are representative of two separate animal runs.

During PCP, the generation of host inflammatory cytokines is important not only for organism clearance but also subsequently for lung impairment. We therefore examined the effects of FcγR gamma chain absence on the production of inflammatory cytokines in host defense against *Pneumocystis*. Accordingly, we investigated the effect of the absence of Dectin-1 and Fc-gamma receptors (FcγRs) on lung cytokine production during PCP. As noted, similar to the H&E slides and lung inflammation scoring noted above and the aberrant *Pneumocystis* clearance, the production of inflammatory cytokines was also significantly reduced in the *Clec7a*^−/−^ *Fcer1g*^−/−^ lungs during PCP. This included significant reductions similar to the *Card9*^−/−^ animals in TNF-α, IL-6, and IL-1β in the CD4-depleted *Clec7a*^−/−^*Fcer1g*^−/−^ compared with WT controls (Fig. 3A through C).

### CLR and FcγR functions together are critical for optimal *Pneumocystis* organism clearance in immune-competent animals

**Fig 4:**
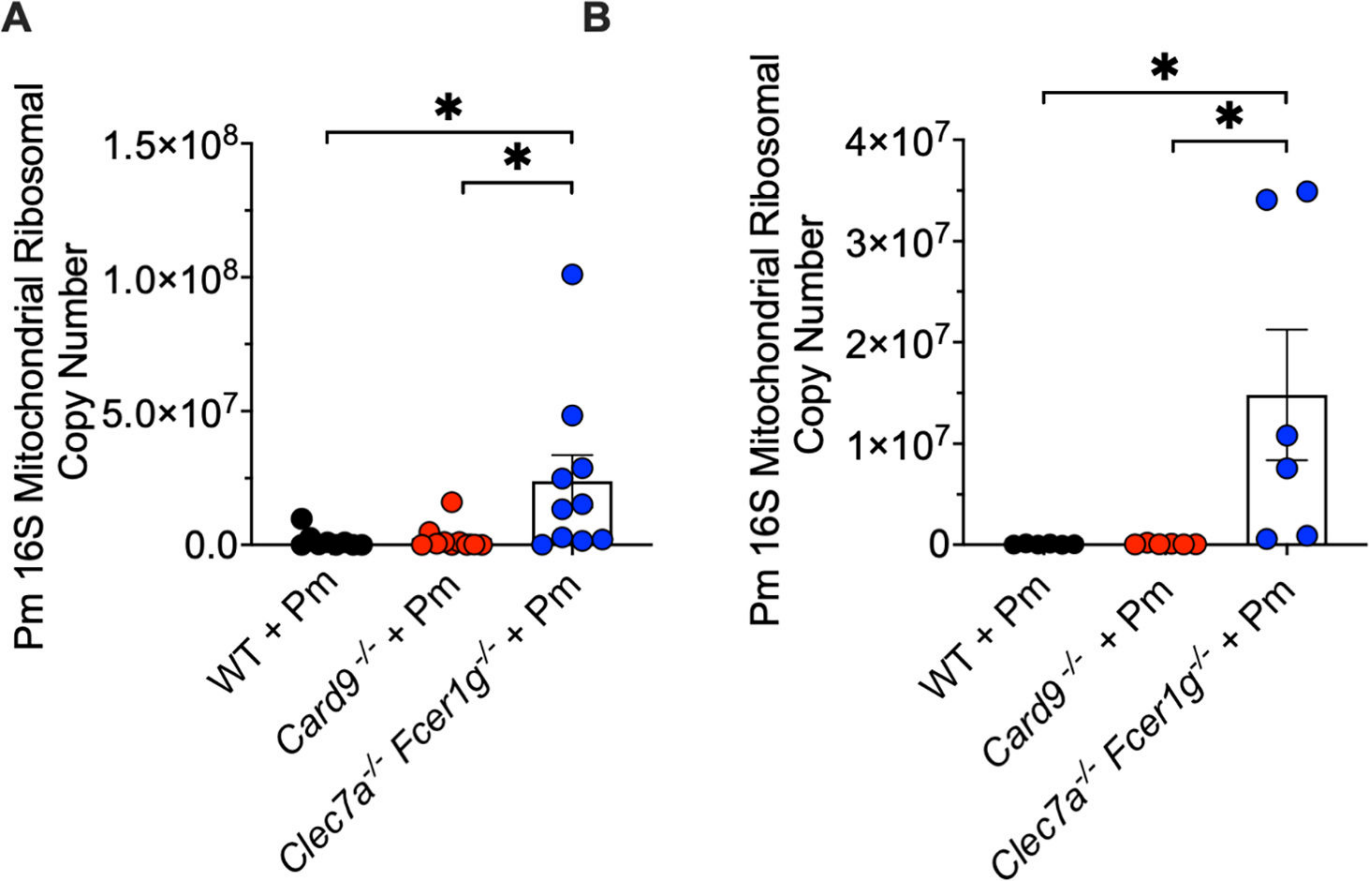
*Clec7a*^−/−^ *Fcer1g*^−/−^ mice are significantly deficient in their ability to clear fungal organisms in an intact T-cell immunocompetent PCP model. WT, *Clec7a*^−/−^ *Fcer1g*^−/−^, or *Card9*^−/−^ immunocompetent mice were infected with Pm. After 30 (**A**) or 60 (**B**) days of infection, the lungs were harvested, and organism burden was determined by *16S mitochondrial ribosomal* DNA–targeted qPCR, and the copy number was assessed using a standard curve. Data in (**A-B**) are derived from *n* > 6 animals per group and shown as mean ± SEM; *P < 0.05.

Next, we wanted to determine if dual deficiencies in CLR signaling and FcγR effector functions affected the clearance of *Pneumocystis* in immunocompetent hosts. Previously, we have reported that in immunocompetent *Card9*^−/−^ animals after 20 days of *Pneumocystis* infection, we noted approximately a 3-fold increase in organism burden compared with WT animals (8). In our current study, we also compared the *Clec7a*^−/−^*Fcer1g*^−/−^ versus *Card9*^−/−^ and WT animals at 30 and 60 days post-infection. As shown in Fig. 4A, after 30 days, we noted that *Card9*^−/−^ animals had similar *Pneumocystis* burdens compared with the WT animals, whereas strikingly, the *Clec7a*^−/−^*Fcer1g*^−/−^ has significantly greater lung organism burden than either the *Card9*^−/-^ or WT animals. Studies extending this infection for 60 days also demonstrate significantly greater organism burden in the *Clec7a*^−/−^*Fcer1g*^−/−^ mice than the other strains, as measured by qPCR (Fig. 4B). To further verify that the absence of FcγRs does not affect normal antibody production/response, we also analyzed the major antibody subclass production by ELISA after 30 and 60 days post-infection in the immunocompetent PCP model. Consistent with prior reports, these mice display normal IgG subclass and IgE production (36). Furthermore, these mice also demonstrated robust IgG subclass PCP-specific responses similar to both WT and *Card9*^−/−^ mice at 30 (Fig. S2A through C) and 60 days (Fig. S2E through G) following infection. In WT mice, IgE production in the PCP model was not significantly increased at both 30 and 60 days post-infection (Fig. S2D and H) and has been reported to not be necessary for PCP host defense (37). These results suggest that the large differences in *P. murina* burden between *Card9*^−/−^and WT versus the *Clec7a^−/−^ Fcer1g^−/−^* mice are not due to defective antibody production but rather to impaired responsiveness to antibody-opsonized organisms.

### CLR and FcγR functions are required for alveolar macrophage inflammatory response to *Pneumocystis*

**Fig 5:**
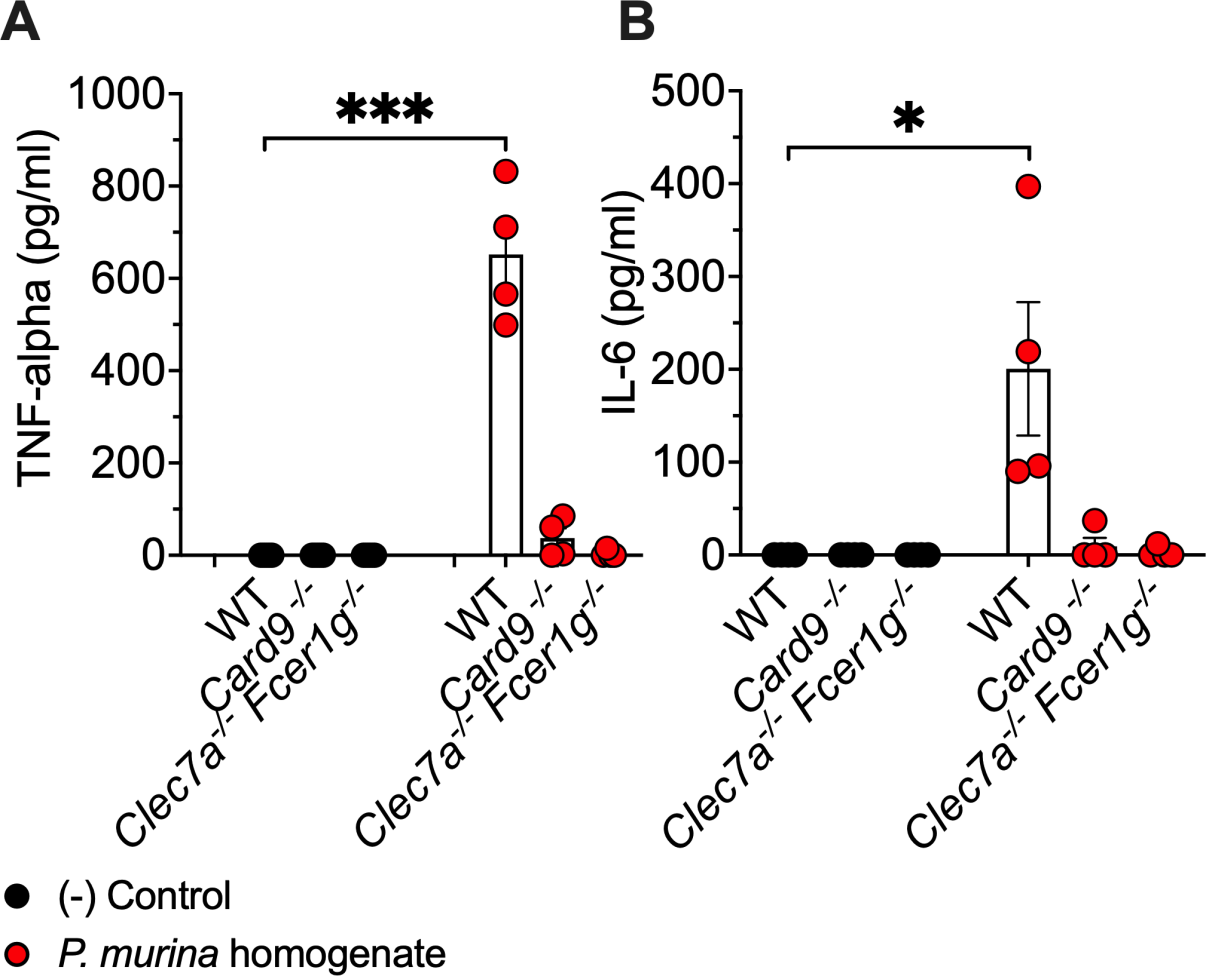
Proinflammatory cytokines are significantly reduced in alveolar macrophage from *Clec7a*^−/−^ *Fcer1g*^−/−^ mice. AMs were stimulated with Pm homogenate for 24 h. After stimulation, macrophage supernatants were collected and analyzed using ELISA for protein levels of (**A**) TNF-α and (**B**) IL-6, respectively. Data shown are integrated means ± the SEM of four independent experiments: *P < 0.05, ***P < 0.001.

Overall, clearance of *Pneumocystis* from the host lung is largely mediated by adaptive CD4 T cell-driven immunity. In immunosuppressed hosts lacking proper T cell function, macrophages also are major effector cells for *Pneumocystis* host defense (13, 38). In that light, we evaluated the potential of isolated alveolar macrophages (AMs) to release proinflammatory cytokines following stimulation with *Pneumocystis in vitro*. We noted that WT AMs secreted significant levels of TNF-α and IL-6 in response to *Pneumocystis* homogenate, but we observed a severe defect in the production of these inflammatory cytokines from both the *Card9*^−/−^ and *Clec7a^−/−^ Fcer1g^−/−^*-deficient macrophages following challenge with *Pneumocystis* (Fig. 5A and B). The degree of reduction of inflammatory cytokines was similar in both the *Card9*^−/−^ and *Clec7a^−/−^ Fcer1g^−/−^*-deficient macrophages.

### CLR and FcγR functions are further required for macrophage differentiation in response to *Pneumocystis*

Similar to our prior published studies of CARD9-deficient bone marrow-derived macrophages (BMDMs), we examined AMs from *Clec7a^−/−^Fcer1g^−/−^* animals for polarization markers including IL-12 (measured via IL-12(p40)) as well as IL-13, both measures of relative M1/Th1 and M2/Th2 macrophage polarization states, respectively, following challenge with *Pneumocystis* (39). Interestingly, ELISA analyses of *Clec7a^−/−^Fcer1g^−/−^* AMs, similar to *Card9*^−/−^ BMDMs stimulated with *Pneumocystis* for 24 h displayed a marked decrease in the expression levels of IL-12(p40) and IL-13, compared with WT-stimulated cells (Fig. S3A and B). These observations suggest that like CARD9 activity in macrophage polarization, CLR and FcγR function also have pronounced effects on macrophage polarization after challenge with *Pneumocystis*.

### Absence of FcγR results in significant reductions in *Pneumocystis* killing in the presence of PCP convalescent serum

**Fig 6:**
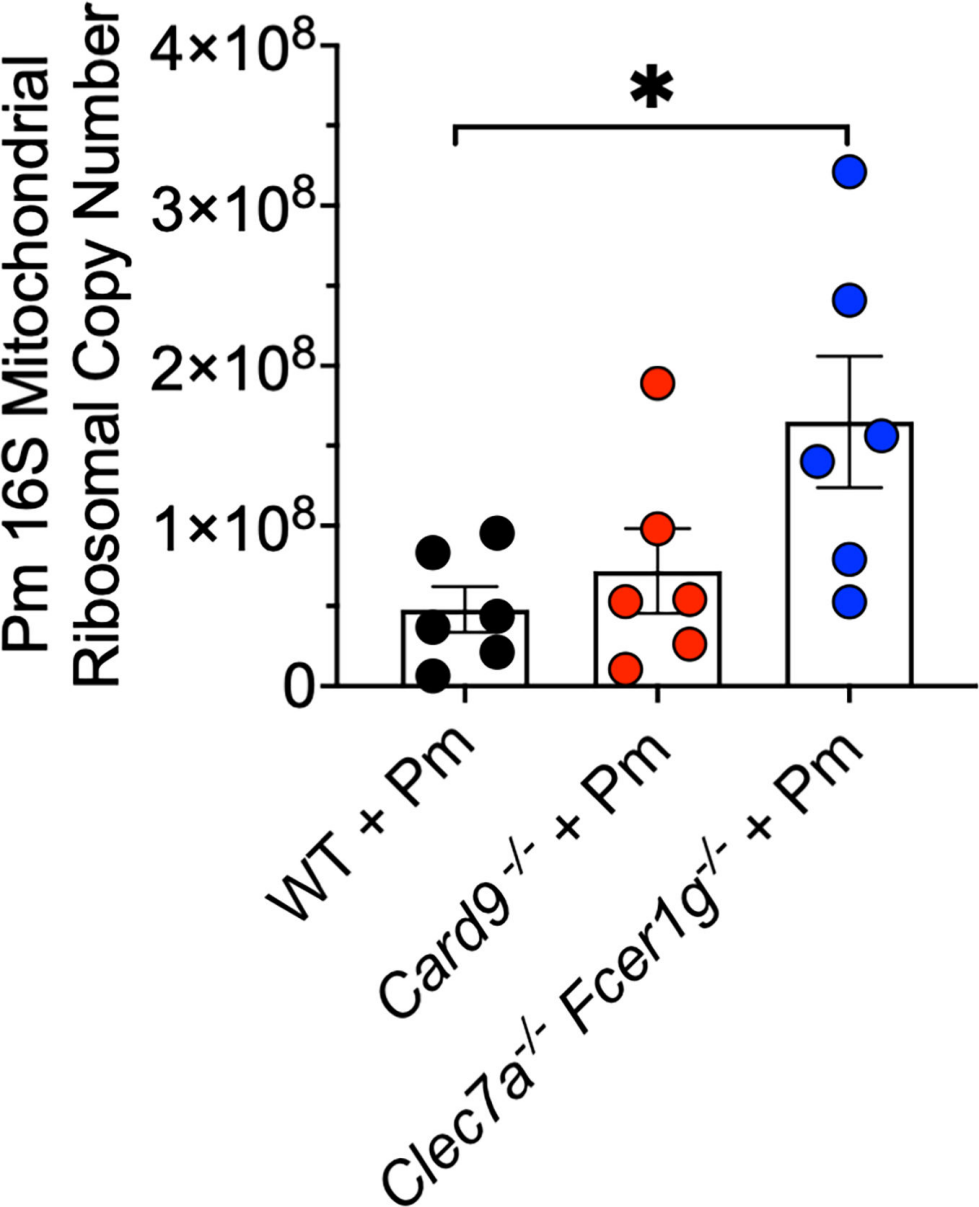
Deficiencies in CLR in and FcγR results in significant reductions in *Pneumocystis* killing in the presence of PCP convalescent (PCPCVS) serum. AMs were stimulated with Pm homogenate for 24 h. Next, fungal organisms were harvested, and organism burden was determined by *16S mitochondrial ribosomal* DNA–targeted qPCR, and the copy number was assessed using a standard curve. Data shown are integrated means ± the SEM of six independent experiments: *P < 0.05.

Although we did not notice significant differences in Ig subclass antibody production between all three groups in the immunocompetent PCP model, we next preincubated AMs *in vitro* with heat-inactivated PCP convalescent serum prior to the addition of live *Pneumocystis* organisms for 18 h. We observed a significant defect of the *Clec7a^−/−^Fcer1g^−/−^* cells in their ability to kill *Pneumocystis* organisms compared with WT cells (Fig. 6). These data suggest that FcγR is not only critical for CLR engagement and downstream proinflammatory signaling but also in opsonization and organism killing.

### The activity of FcRγ is dominant over Dectin-1 for the clearance of organism burden

**Fig 7:**
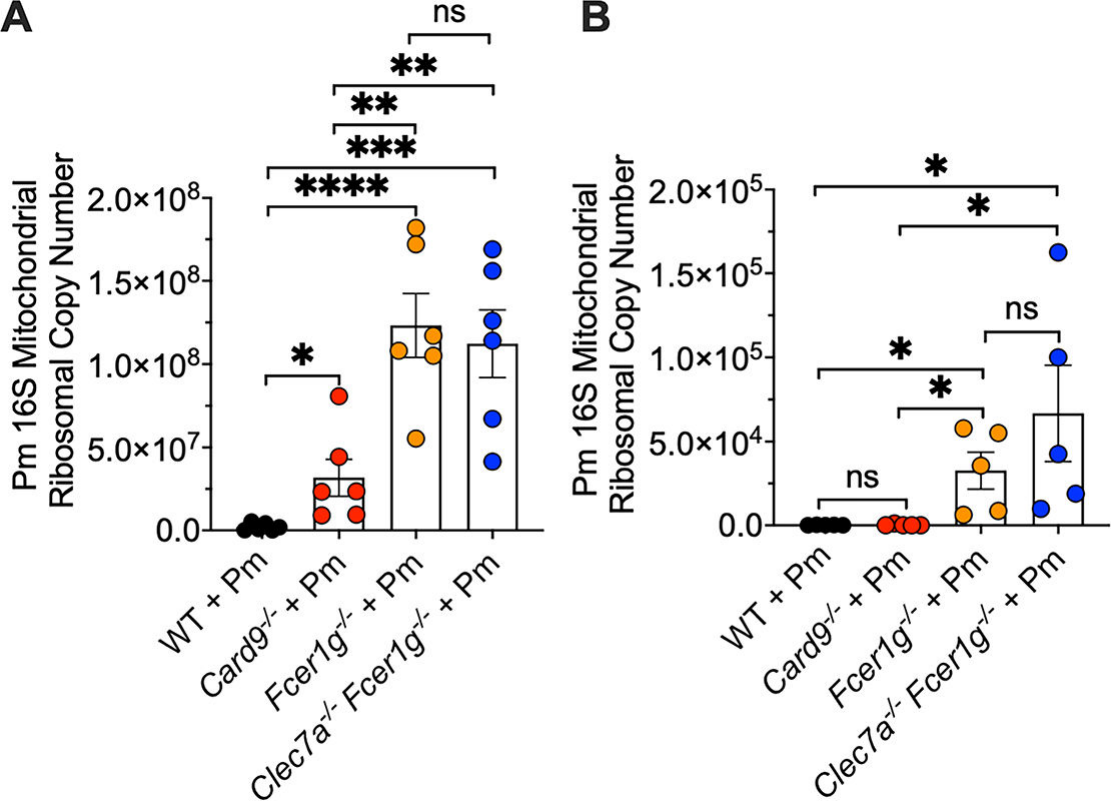
In the absence of FcRγ, Dectin-1 (Clec7A) does not have significant independent effects on *Pneumocystis* burden. Similar defects in Pm killing in the *Fcer1g*^−/−^ and *Clec7a*^−/−^*Fcer1g*^−/−^ animals in both the immunosuppressed (**A**) and 30-day immunocompetent (**B**) PCP models as measured by qPCR utilizing the *16S mitochondrial ribosomal* DNA. Data are derived from 5 to 6 mice per group and shown as mean ± SEM; *P < 0.05, **P < 0.01, ***P < 0.001, and ****P < 0.0001 comparing Pm burden in the *Card9*^−/−^, *Clec7a*^−/−^ *Fcer1g*^−/−^, and WT mice and are representative of two separate experiments.

We next sought to determine whether the activity of FcRγ was dominant over the Dectin-1 CLR activity with respect to organism clearance. To evaluate this, we further compared the *Clec7a^−/−^Fcer1g^−/−^* and *Card9*^−/−^ mouse strains with the *Fcer1g^−/−^* strain lacking only the FcRγ chain in both the CD4-depleted immunocompromised (Fig. 7A) and 30-day immunocompetent PCP mouse models (Fig. 7B). Interestingly, we observed very similar *Pneumocystis* burdens in the *Fcer1g^−/−^* single mutant and the *Clec7a^−/−^Fcer1g^−/−^* double-deficient mouse strains in both the immunocompromised and immune-competent *P. murina* infection models (Fig. 7A and B). These data suggest that FcRγ activity exerts a dominant effect over Dectin-1 activity in the clearance of *P. murina* burden. Thus, FcRγ plays an important role in controlling *Pneumocystis* burden, beyond its role as an inflammatory cofactor engaging with CLRs for inflammatory signaling.

### Blockage of FcRγ with specific monoclonal antibodies reduces alveolar macrophage TNF-α response to *Pneumocystis*

**Fig 8:**
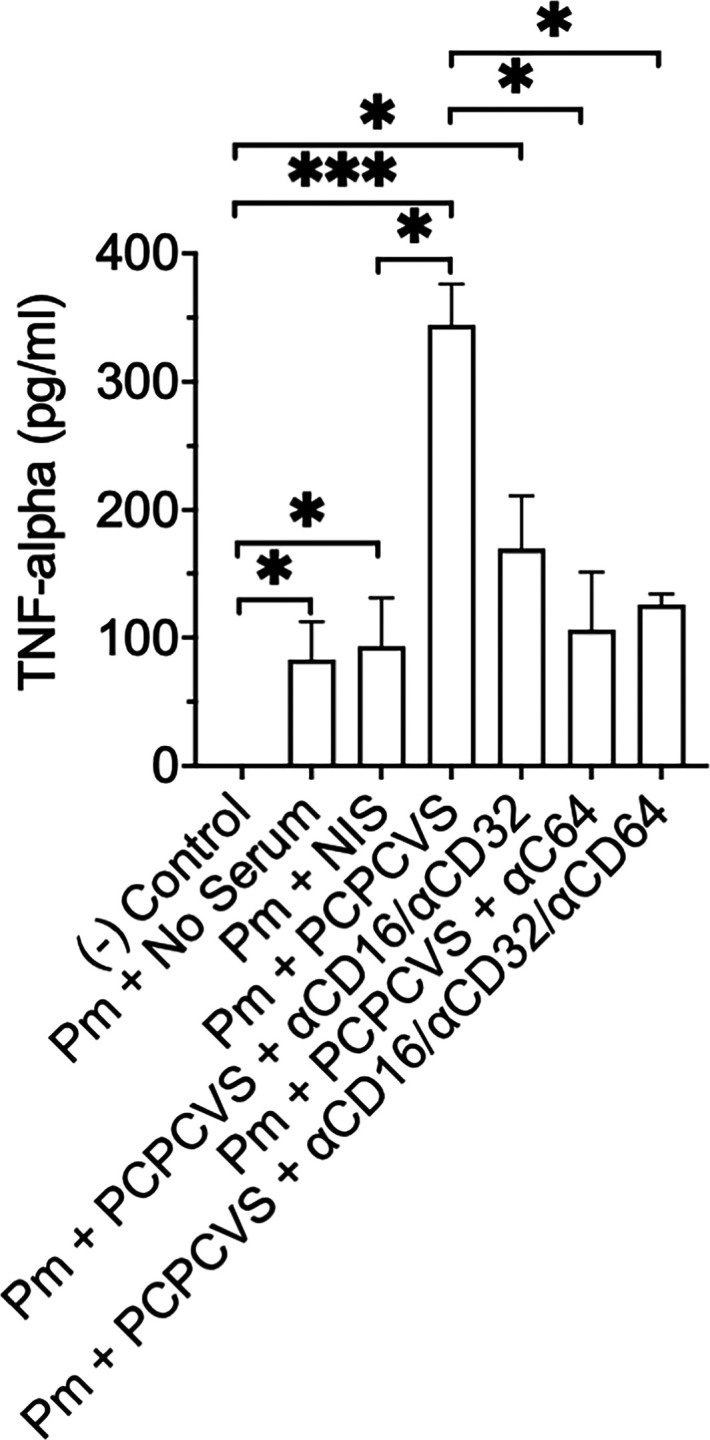
Antibody blockage of specific FcγRs in alveolar macrophages stimulated with *Pneumocystis* results in significant reductions in the proinflammatory response. Additions of monoclonal antibodies against the respective FcγR’s for 1 h prior to the addition of *P. murina* (Pm) for 18 h results in significant decreases of TNF-α in AMs. After stimulation, the supernatants were harvested and analyzed by ELISA for TNF-α protein. The negative control consists of mouse alveolar macrophages alone, with no *P. murina* or serum. Data shown are integrated means ± the SEM of three independent experiments: (*P < 0.05, **P < 0.01, ***P < 0.001).

We have shown that AMs FcRγs (CD16, CD32, or CD64) are critical for the killing of opsonized *P. murina* fungal organisms in the presence of convalescent serum (Fig. 6) from PCP mice. Therefore, we analyzed the specific role of FcRγs (CD16, CD32, CD64, or in combination) in AMs for TNF-α responses by blockade of these receptors with specific monoclonal antibodies during overnight challenge with *P. murina* in culture. PCP convalescent serum (PCPCVS) incubated with *P. murina* (Fig. 8, lane 4) resulted in robust inflammatory macrophage TNF-α response compared with no serum or non-immune (NIS) serum challenged with organisms. In addition, aCD64 alone (lane 6) or in combination with aCD16/aCD32 (lane 7) resulted in significant reductions in TNF-α release from AMs incubated with *P. murina* demonstrating the role of these FcRγs in alveolar macrophage inflammatory responses to *P. murina*.

### CARD9 inhibition reduces lung inflammation during *Pneumocystis* infection

**Fig 9:**
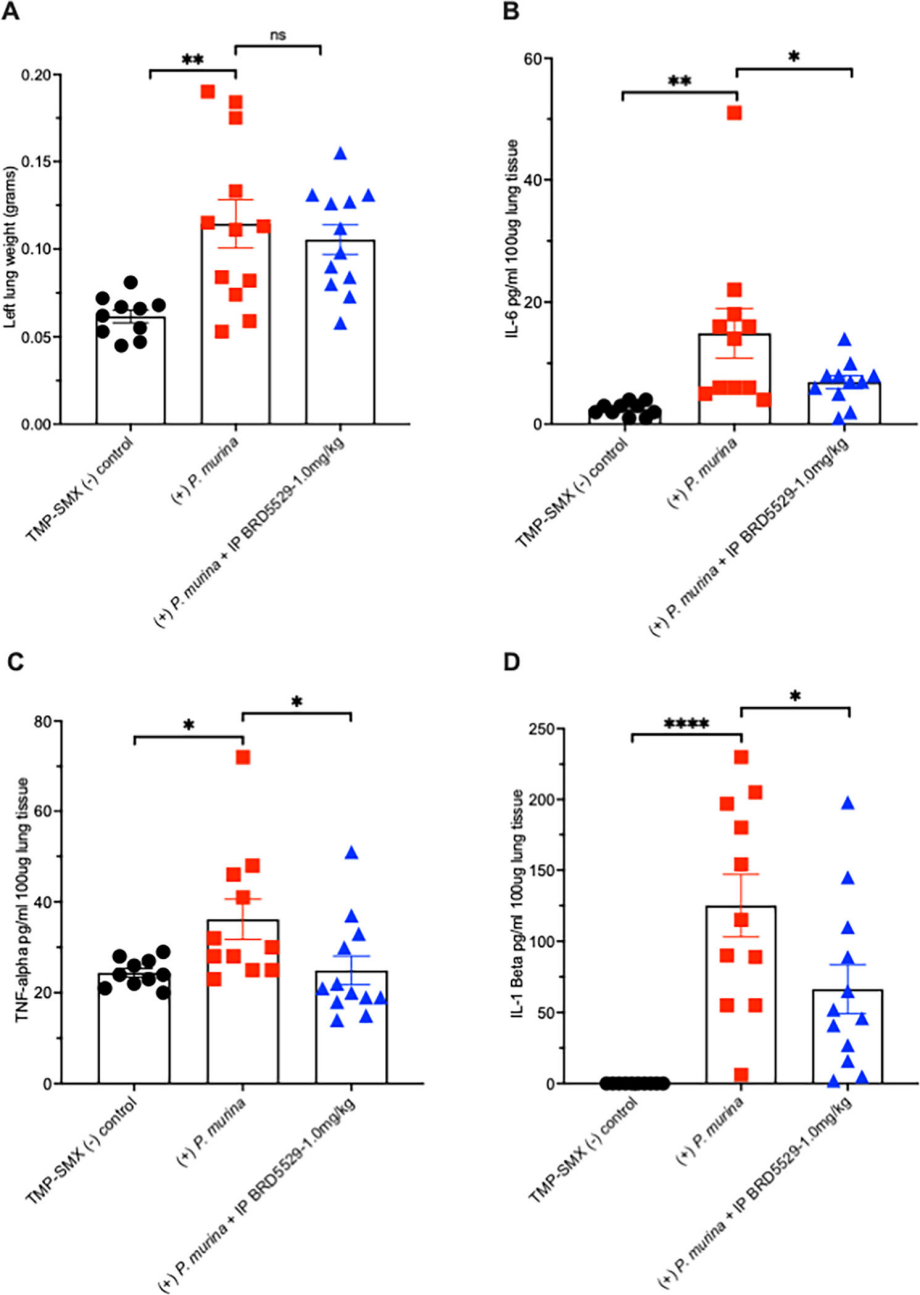
Inhibition of CARD9 results in reduced lung inflammation during *Pneumocystis* Pneumonia. Mice were depleted of CD4 lymphocytes and inoculated with *P. murina*. Following 7 weeks of infection, the mice were treated with BRD5529 (1.0 mg/kg), a small molecule inhibitor of CARD9, or with vehicle via IP injection daily for 10 days. Additional mice were maintained on trimethoprim sulfamethoxazole to eliminate infection. Shown are (**A**) left lung weights (grams), (**B**) IL-6 (pg/mL, per 100 mg of lung tissue), (**C**) TNF-a (pg/mL, per 100 mg of lung tissue), and (**D**) (IL-1b, pg/mL, per 100 mg of lung tissue). (* Denotes *P* < 0.05, **P < 0.005, and ****P < 0.04).

Our work further underscores the role of CLR signaling through CARD9 in mediating lung inflammation during *Pneumocystis* pneumonia, which can promote respiratory impairment during severe disease. We have prev iously shown that a small molecule, termed BRD5529, an inhibitor of CARD9, can reduce macrophage inflammatory signaling responses to *Pneumocystis* b-glucans (28), but this agent has never been tested in a whole animal infection model (27). Accordingly, we assessed the effect of BRD5529 in the CD4-depleted *P. murina* pneumonia model (Fig. 9). Although lung weights (used previously to measure lung injury in mice [40, 41]) were only modestly reduced by BRD5529 treatment (Fig. 9A), we observed significant reductions in IL-6 (Fig. 9B), TNF-a (Fig. 9C), and IL-1b (Fig. 9D) in the *P. murina*-infected lung following BRD5529 treatment compared with the vehicle alone control with *P. murina* pneumonia. These data suggest that CARD9-mediated lung inflammation can represent an independent target for reducing exuberant lung inflammation during severe *Pneumocystis* pneumonia.

## DISCUSSION

Our report highlights the critical contributions that both CLRs as well as FcRγs make in *Pneumocystis* clearance and host inflammatory responses during *Pneumocystis* pneumonia. We demonstrated that the combined absence of both CLR signaling as well as FcRγ function results in substantially increased organism burden as well as significant reductions in lung inflammatory responses. Our studies concurrently underscore the importance of both CLR-CARD9-mediated and humoral responses in controlling this infection. With mortality of *Pneumocystis* pneumonia ranging as high as 50%, we strongly believe that a better understanding of host defenses to this organism is critical (42). Furthermore, additional strategies to assist in the management of PJP are also clearly needed. Recently, the World Health Organization (WHO) listed *P. jirovecii* among the priority fungal pathogens for research, development, and public health action (43).

The absence of CARD9 results in various infection outcomes depending on the pathogen and the organ or compartment of infection (8, 29). For instance, the absence of CARD9 in mouse brain results in significantly increased *C. albicans* burdens (30). To phenocopy CARD9 deficiency, these researchers also used the *Clec7a^−/−^Fcer1g^−/−^* mouse line that we employed in our current study. These mice lack Dectin-1 (*Clec7a^−/−^*), with its cognate ITAM domain for cell signaling, as well as FcRγ that Dectin-2, Dectin-3, and Mincle use to activate cell signaling. In their study of *C. albicans* brain infection, the *Clec7a^−/−^Fcer1g^−/−^* mice displayed similar fungal burdens to *Card9*^−/−^ mice, replicating the CARD9 deficiency result (30). However, in the case of *Pneumocystis* pneumonia, our results were quite different with significantly greater *P. murina* organism burdens being observed in the double-deficient mice compared with *Card9*^−/−^ single-deficient animals. The differing outcomes of various infection models may reflect organism-specific issues, as well as the local organ immune microenvironments at the site of infection.

Innate immune recognition through CLRs including Dectin-1, Dectin-2, and Mincle represent important mechanisms of host immune signaling during infection with *Pneumocystis* (24, 44–46). Following organism engagement with these receptors, downstream Syk activation occurs, and CARD9 assembly with BCL-10 and MALT1 triggers MAPK and NF-κB activation, resulting in inflammatory cytokine generation (47). We have demonstrated the importance of the CARD9 pathway in PCP as well as the ability to target the protein with a specific small molecule inhibitor (termed BRD5529), resulting in a reduction in proinflammatory responses to fungal β-glucan challenge both *in vitro* and *in vivo* (27, 28). In the current study, we further document that BRD5529 can significantly reduce lung inflammation in a mouse model of active PCP, providing further support that targeting CARD9 may represent an additional therapeutic approach to blunt exuberant and deleterious lung inflammation during severe *Pneumocystis* pneumonia (48).

Although the targeting of CLR-CARD9 pathways represents a promising therapeutic approach to treating lung inflammation associated with PCP, additional alternative strategies are also needed for killing fungal organisms. Although current treatments including trimethoprim and sulfamethoxazole (TMP-SMX) are effective and relatively inexpensive (49, 50), concerns remain about serious adverse events, including renal and hematological complications (42). In addition, due to the risk of bone marrow suppression associated with TMP-SMX, clinicians are often hesitant to use this medication in stem cell transplant patients (51). Likewise, other treatments for PJP, including atovaquone, dapsone, and aerosolized pentamidine can be effective but are associated with variable gastrointestinal adsorption and drug resistance (52, 53). Other investigations indicate that in addition to antibiotics, the use of antibodies directed against the organism may also enhance elimination of this infection. For example, immunoglobulin M monoclonal antibodies recognizing *Pneumocystis* were protective and reduced organism numbers by greater than 99% (54–56). Other studies further show that B-cell depletion may be a risk factor for *Pneumocystis* pneumonia (18). Such function of B-cells in PCP is at least in part mediated through CD40 activity of the B cells (20).

The central role of CARD9 has been clearly demonstrated in several other fungal pathogen/host immune interactions (8, 29, 57–62). Our current study underscores that the CLR-CARD9 pathway as well as that FcRγs strongly participate in host inflammatory responses to *Pneumocystis* as well as organism killing within the lung. Indeed, when PCP convalescent serum was added to the AMs lacking FcγRs (compared with wild-type and *Card9*^−/−^ AMs), the macrophages demonstrated significant impairment of *Pneumocystis* killing, indicating the importance of FcγR antibody-mediated interaction with *Pneumocystis*. These data further support the possible therapeutic use of antibody-based strategies for individuals with PJP failing antibiotics alone. In addition, such strategies can also be combined with inhibition of CARD9 signaling to concurrently control exuberant and injurious lung inflammation during *Pneumocystis* pneumonia.

## MATERIALS AND METHODS

### Animals

Mice with a targeted mutation in CARD9 (*Card9^−/−^*) or CLEC7A FCER1G (*Clec7a^−/−^ Fcer1g^−/−^*) on the C57BL/6 background were described previously (8, 30). Wild-type C57BL/6 mice were purchased from Charles River Laboratories (Wilmington, MA). Approximately equal numbers of both male and female mice aged 6–10 weeks were used in all experiments.

### Isolation of *Pneumocystis*

*P. murina* organisms were derived from the American Type Culture Collection and propagated through the *Rag2^tm1Fwa^Il2rg^tm1Wjl^* mice (purchased from Taconic Biosciences) (63, 8). Animals were infected with *P. murina* after 8 weeks of infection, and organisms were harvested as previously described (44, 64).

### Murine models of infection

Mice from *Card9^−/−^*, *Clec7a^−/−^ Fcer1g^−/−^*, and wild-type strains were inoculated with 1 × 10^5^ *P. murina* nuclei intratracheally. For the immunocompetent infection models, mice were sacrificed either at 30 or 60 days after infection (8, 44).(65) To establish the PCP-immunosuppressed animal model, wild-type, *Card9^−/−^*, and *Clec7a^−/−^ Fcer1g^−/−^* mice received weekly intraperitoneal injection (IP) of GK1.5 (0.3 mg) antibody to deplete CD4 T cells (66). After 14 days, the mice were anesthetized (ketamine) and inoculated with 1 × 10^5^ *P. murina* intratracheally. Finally, the mice were sacrificed 8 weeks after infection. Five to 10 animals per group were used in each experiment, and *P. murina* burdens and inflammatory markers were assayed on lung tissues using ELISA and qPCR.

### AM isolation and culture

AM isolation was conducted as previously described (67). Briefly, mouse lungs were lavaged with sterile calcium and magnesium-free Dulbecco's Minimal Essential Medium (DMEM) with 0.5 mM Ethylenediaminetetraacetic acid (EDTA) supplement. AMs isolated from the lavage fluid were pelleted by centrifugation at 300 × *g* at 4°C and subsequently resuspended in Roswell Park Memorial Institute (RPMI) 1640 medium and 10% fetal bovine serum with antibiotics. AMs were enumerated using a hemocytometer and plated in duplicate at a cell density of 2 × 10^5^/well in a 96-well plate.

### ELISA determination of cytokine release

Cytokines were analyzed from the cell culture supernatants or the total lung homogenates. ELISA kits to measure mouse TNF-α, IL-13, IL-12(P40), IL-6, and IL-1β were purchased from ThermoFisher Scientific, Waltham, MA.

### Analysis of β-1,3 glucan content in lungs in the immunosuppressed model

Briefly, mouse lungs from the immunosuppressed PCP mouse model as described above were harvested. A TissueLyser LT (Qiagen, Germantown, MD) was used to create lung lysates (8). In total, 100 μg of total lung protein was plated in duplicate wells, and quantification of β−1,3 glucan in the infected lungs was conducted with the Glucatell® assay kit (Associates of Cape Cod, Ind., East Falmouth, MA).

### ELISA determination of *Pneumocystis* major surface glycoprotein (Msg)

Similar to as stated above, 100 μg/well total lung lysate in duplicate wells was plated overnight at 4°C. The following day, the plates were washed 4× with 1× PBS/0.05% Tween 20 (PBS/Tween). Plates were then blocked with 1× PBS/Tween plus 3% bovine serum albumin (BSA) for 1 h at RT. The plates were then washed 4× with PBS/Tween. Next, a 1:5,000 dilution of an antibody generated against *P. murina* major surface glycoprotein (Msg) (33) in 1× PBS/Tween/3% BSA was added at RT for 1 h. The plate was washed again as stated above 4×, and a 1:6,000 dilution of goat anti-rabbit Ig-HRP (in 1× PBS/Tween/3% BSA) was added to the plate for 1 h at RT. The plate was washed again as noted, and a 1:250 dilution of streptavidin (ThermoFisher Scientific) in 1× PBS/Tween/3% BSA was added for 30 min at RT. The plates were washed again as described, and a 1:250 dilution of avidin-HRP (ThermoFisher Scientific) was added to the plate for 15 min at RT. After washing the plates six times, 3,3'5,5'-tetramethylbenzidine (TMB) substrate was added for 15 min at RT to stop the reaction, 50 mL/well of 2.0 N H_2_SO_4_ was added, and the plates were read at 450 nm using a VersaMax plate reader (Molecular Devices, San Jose, CA). A standard curve of Msg from 4 μg to 500 μg per well was used for the quantification of Msg content in the lungs.

### *Pneumocystis* serum IgG and IgE ELISAs

Anti-*P*. *murina* serum IgG2a, IgG2b, IgG3, and IgE levels were measured by using a sandwich ELISA. Fungal lysates (1.0 mg/well in 0.1M sodium bicarbonate buffer, pH 8.3) were adsorbed onto 96-well microtiter plates by incubating at 4°C overnight. The plates were washed four times with PBS/Tween. Next, the plates were blocked for 1 h at RT with PBS/Tween 1% BSA blocking buffer (Pierce). The plates were then washed four times, and the sample mouse sera, diluted 1:10, was added to the wells and incubated at RT for 2 h. The plates were washed four times, and 100 μg/well of the respective HRP-conjugated goat anti-mouse Ig subclass or IgE (SouthernBiotech, Birmingham, AL) at 1:4,000 in PBS/Tween 1% BSA blocking buffer was added for 1 h at RT. Finally, after washing the plates six times, TMB substrate was added for 15 min at RT, followed by stopping the reaction with 2.0 N H_2_SO_4_. The plates were read at 450 nm using a VersaMax plate reader.

### Quantitative qPCR

Alveolar macrophages or lung tissues were lysed using QIAshredders and TissueLyser LT, respectively, both purchased from Qiagen. Total RNA was purified using the RNeasy minikit (Qiagen). An iScript Select cDNA synthesis kit (Bio-Rad, Hercules, CA) was used for reverse transcription using oligo (dT) primers and a random hexamer primer mix. A SYBR green PCR kit (Bio-Rad) was used for quantitative real-time PCR and was performed and analyzed on a CFX Connect PCR machine (Bio-Rad). The sequences of the primer pairs are listed in Table S1. Pm 16S Mitochondrial Ribosomal Copy Number (mean) for the qPCR quantification of mouse lung organism burden is listed in Table S2.

### Determination of *Pneumocystis* burden

After sacrifice, the right lung was flash frozen for the quantification of *Pneumocystis* burden using qPCR. DNA was isolated using the DNeasy Blood and Tissue kit (Qiagen). A qPCR assay was performed to enumerate *P. murina* organism burden with primers to Pm *16S mitochondrial ribosomal RNA* DNA (Table S1). Amplification of lung cDNA samples was compared with a standard curve, and the resulting measure of DNA copies was proportioned to the net *P. murina* burden. To quantify live *Pneumocystis* organisms in cell cultures, total RNA was isolated from the entire contents of the well and converted to cDNA using the iScript Select cDNA synthesis kit (Bio-Rad, Hercules, CA), and similar procedures were followed using primers to the *16S mitochondrial rRNA* DNA.

### FcγR blocking experiments

FcγR blocking experiments were conducted on mouse alveolar macrophages as previously described (68). Briefly, alveolar macrophages were isolated and plated in 96-well plates as described above. After 2 h, the macrophages were pre-incubated for 1 h with 10 μg/mL with one of the following: nonimmune serum from uninfected C57/BL6 mice, PCP convalescent serum (PCPCVS) from 8- to 10-week *P. murina*-infected mice, rat anti-mouse CD16/32 (BD Biosciences), mouse anti-mouse CD64 (Biolegend), or a combination of both antibodies at 10 μg/mL. Following the pre-incubation, *P. murina* organisms were added at a multiplicity of infection (MOI) of 10:1 and incubated overnight at 37°C/5% CO_2_. The supernatants were then harvested and analyzed for TNF-alpha release by ELISA, as previously described (69).

### Immunohistochemistry

Paraffin embedding and staining were performed at the Mayo Clinic Histology Core, Scottsdale, AZ. Right and left lungs were inflation-fixed with 10% buffered formalin overnight and embedded in paraffin. Sections (5 mm) were stained with Gomori Methenamine Silver stain (GMS) and H&E.

### Histology analysis

Lung samples were preserved in 10% neutral formalin. Paraffin embedding and staining were conducted at the Mayo Clinic Histology Core in Scottsdale, AZ, USA. Tissue sections (5 µm) were stained with H&E and evaluated blindly by a Mayo Clinic pathologist for the degree of lung inflammation (28). The inflammation was scored as follows: 1+ indicated mild perivascular aggregates; 2+ indicated heavy perivascular aggregates; 3+ indicated mild alveolar aggregates; 4+ indicated alveolar exudate and heavy alveolar aggregates; and 0 indicated normal tissue. These semi-quantitative scores were determined based on the entire organ surface area present in the slide section. In addition, overall lung cellularity was also scored using an adaptation of the method of Fulmer et al. (70). Grading of both the degree of cellularity was made with an estimate of the increase in each compared with normal lung. The grade +1 was assigned when there was a <25% increase; grade +2 represented 25%–50% increase; grade +3 was assigned when there was a 50%–75% increase; and grade +4 was assigned when there was a >75% increase.

### Effect of CARD9 inhibition on lung inflammation during *Pneumocystis* infection

Wildtype mice were CD4-depleted with GK1.5 and inoculated with *P. murina* as described above. After 7 weeks of infection, mice received BRD5529 (1.0 mg/kg) or vehicle control. As we previously reported, BRD5529 lacks solubility in water or saline. Therefore, the BRD5529 was prepared in 1% Methocel (28). IP treatment of BRD5529 (1.0 mg/kg) in 1% Methocel vehicle or vehicle alone was administered daily to the infected mice for 10 days. Additional control mice were maintained on TMP-SMX in the drinking water to prevent infection. After 10 days of therapy, the lungs were harvested, and the proteins were extracted for assessment of lung inflammation by ELISA.

### Statistical analysis

Statistical differences between various experimental conditions were first assessed using ANOVA and then by Student’s *t* tests as indicated. Nonparametric statistics were used when the data were distributed in a non-Gaussian manner. Statistical testing was performed using GraphPad Prism version 10.2.2 software, and statistical differences were considered significant when *P* < 0.05 or greater.

## SUPPLEMENTAL MATERIAL

Supplemental material iai.00276-24-s0001.docx Table S1; Fig. S1 to S3.

ASM does not own the copyrights to Supplemental Material that may be linked to, or accessed through, an article. The authors have granted ASM a non-exclusive, world-wide license to publish the Supplemental Material files. Please contact the corresponding author directly for reuse.
