# Supplementary material for: The importance of Fcγ and C-type lectin receptors in host immune responses during Pneumocystis pneumonia
Source: Infect Immun. 2024 Dec 31;93(2):e00276-24. doi: 10.1128/iai.00276-24 (PMC11834440; doi:10.1128/iai.00276-24)
Supplement: Supplemental material — Table S1; Fig. S1 to S3. [file iai.00276-24-s0001.docx]

**Supplementary Material for**

**The Importance of Fcg and C-Type Lectin Receptors in Host Immune Responses During**

***Pneumocystis* Pneumonia**

**Supplementary Table 1**

| **Gene Name** | **Forward primer** | **Reverse primer** |
| --- | --- | --- |
| *B2m* | CTCGGTGACCCTGGTCTTTC | GGATTTCAATGTGAGGCGGG |
| *IL-1*β | AATCTCGCAGCAGCACATC | CACACACCAGCAGGTTATC |
| *IL-6* | GCAAGAGACTTCCATCCCG | TCATTTCCACGATTTCCCAG |
| *TNF-α* | GTCTCAGCCTCTTCTCATTC | TTGGTGGTTTGCTACGACG |
| *Sp* | AGTAGGTGTCTCGTCACATAAAG | CTGGAAGGGTTGAGTATCATAGAG |
| *Gsc1* | GGGGAATATTATGCGCCGGA | GCTGATCCGCCATAGGAACA |
| *16S mitochondrial ribosomal RNA* | GATGGCTGTTTCCAAGCCCA | GTGTACGTTGAAAGTACTC |

**Supplementary Figure 1.**

**
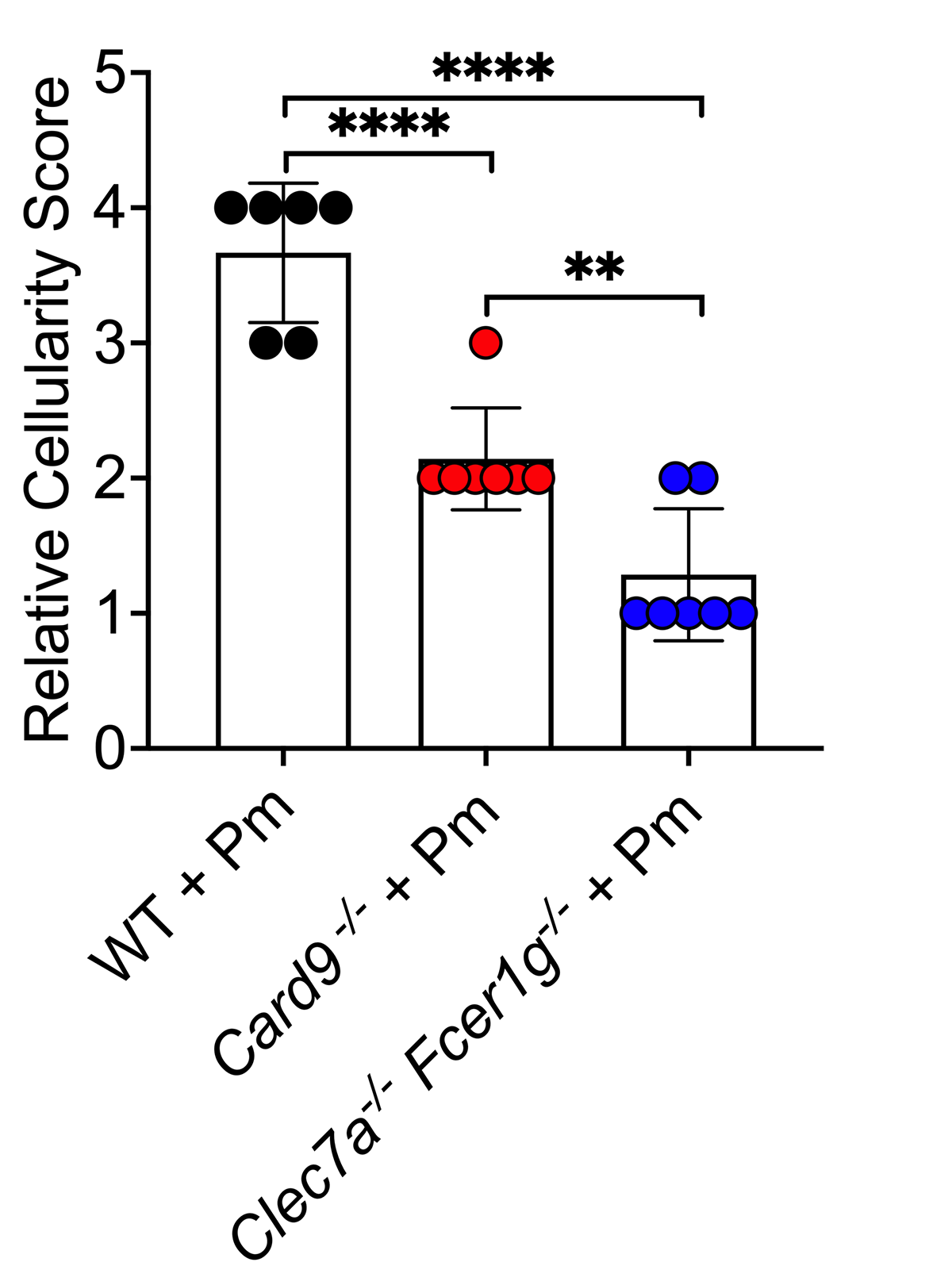
**

**Supplementary Figure 1. Lung cellularity was significantly reduced in *Card9^-/-^* and *Clec7a*^−/−^ *Fcer1g*^−/−^ *P. murina* infected mice compare to wildtype infected mice.** WT, *Clec7a*^−/−^ *Fcer1g*^−/−^, or *Card9^-/-^* CD4 depleted mice were infected with *P. murina* (Pm). After 8 weeks of infection, the lungs were fixed in 10% phosphate-buffered formalin and 5-µm sections were obtained and stained with hematoxylin and eosin. Lung cellularity scores were obtained as described under methods. The Pm infected *Clec7a*^−/−^ *Fcer1g*^−/−^ and *Card9^-/-^* mice showed significantly reduced cellularity compared to Pm infected wildtype mice. In addition, the *Clec7a*^−/−^ *Fcer1g*^−/−^ mice exhibited even less lung cellularity compared to *Card9^-/-^* mice. **p < 0.01 and ****p < 0.0001 comparing the denoted Pm infected group. The data shown are derived from 6 animals and shown as mean + SEM per group.

**Supplementary Figure 2.**

**
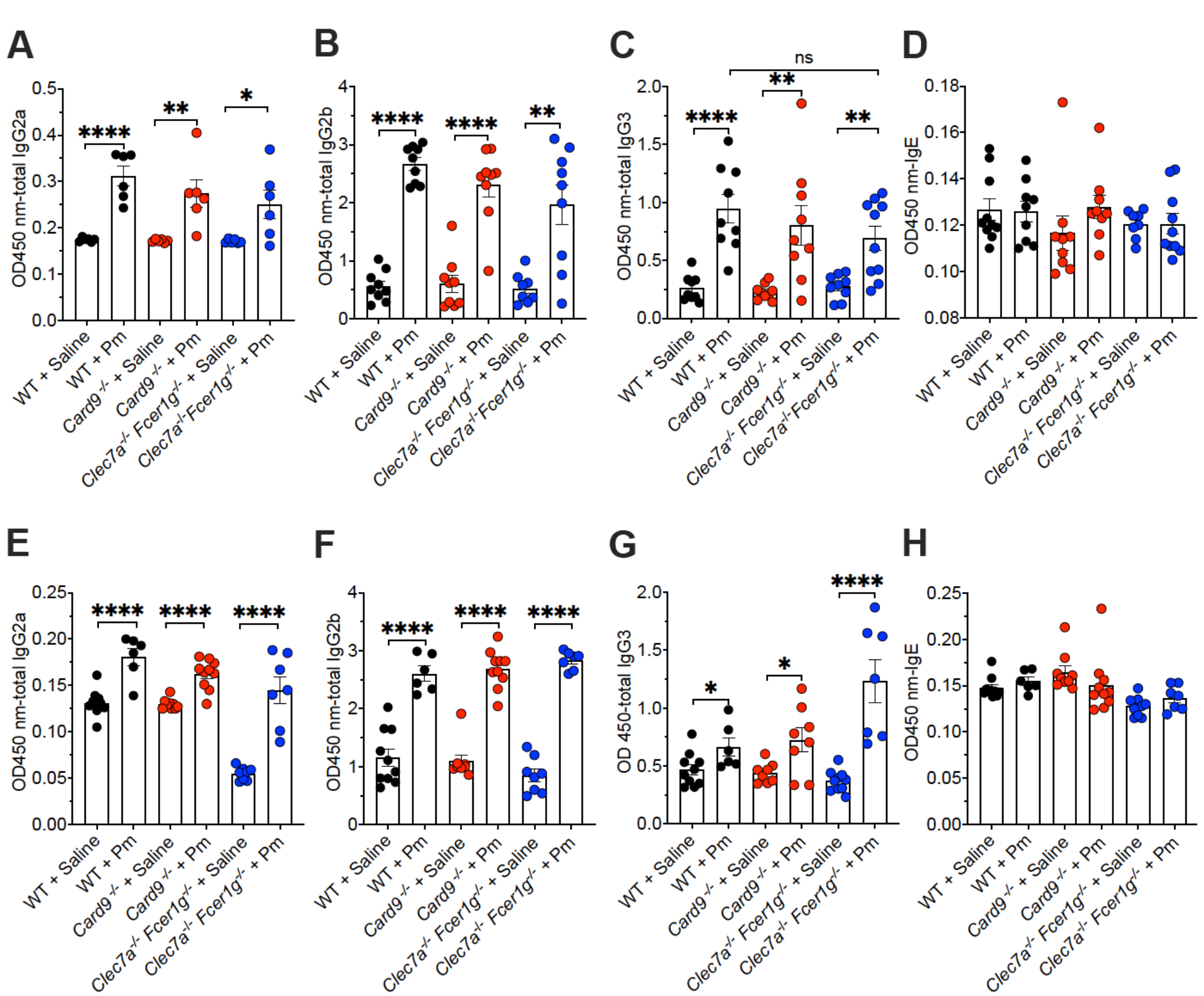
**

**Supplementary Figure 2. WT, *Clec7a*^−/−^ *Fcer1g*^−/−^, and *Card9^-/-^* *P. murina* infected mice all generate significant and similar IgG subclass antibody subclasses.** WT, *Clec7a*^−/−^ *Fcer1g*^−/−^, or *Card9^-/-^* immunocompetent mice were infected with *P. murina* (Pm). After 30 **(A-D)** or 60 **(E-H)** days of infection, serum was collected and ELISAs performed for the listed Ig antibody subclasses or IgE antibody. **p* < 0.05, **p < 0.01, and ****p < 0.0001 comparing the respective saline versus its Pm infected group. The data shown are derived from 6 animals and shown as mean + SEM per group.

**Supplementary Figure 3.**

**
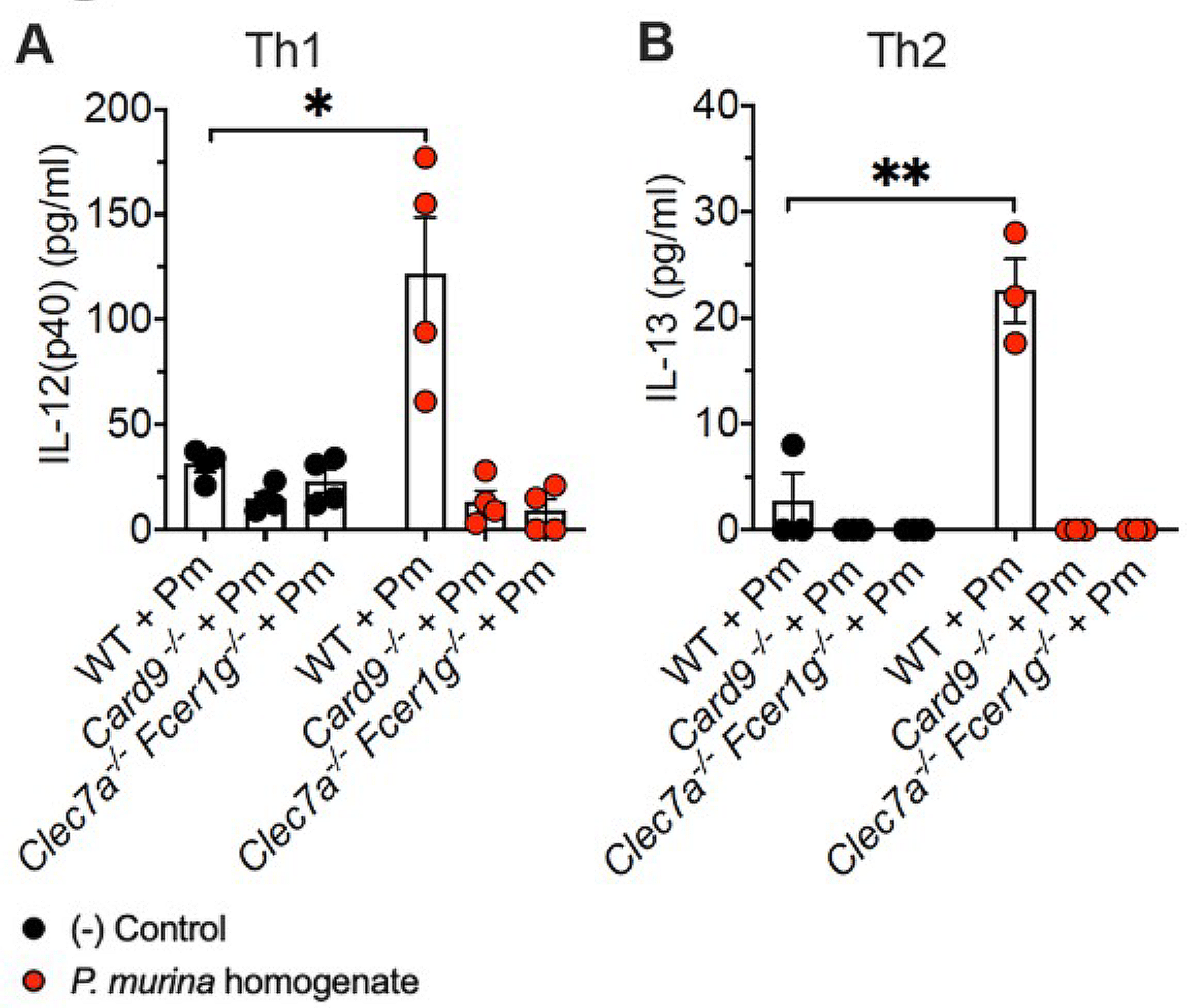
**

**Supplementary Figure 3. Similar to CARD9, alveolar macrophages require CLR and FcγR functions for polarization in response to *Pneumocystis*.** AMs were stimulated with *P. murina* (Pm) homogenate for 24 hr. After stimulation, macrophage supernatants were collected and analyzed by ELISA for protein levels of M1 **(A)** IL-12(p40) and M2 **(B)** IL-13 markers, respectively. Data shown are integrated means ± SEM of three to four independent experiments: **p* < 0.05, p** < 0.01.
